# Acute Effects of Exposure to a Traditional Rural Environment on Urban Dwellers: A Crossover Field Study in Terraced Farmland

**DOI:** 10.3390/ijerph120201874

**Published:** 2015-02-05

**Authors:** Juyoung Lee, Bum-Jin Park, Tatsuro Ohira, Takahide Kagawa, Yoshifumi Miyazaki

**Affiliations:** 1 Korea Forest Service, Government Complex 1, 189 Cheongsa-Ro, Seo-Gu, Daejeon 302-701, Korea; E-Mail: lohawi@gmail.com; 2 College of Agriculture and Life Sciences, Chungnam National University, 99 Daehak-Ro, Yuseong-Gu, Daejeon 305-764, Korea; E-Mail: bjpark@cnu.ac.jp; 3 Forestry and Forest Products Research Institute, 1 Matsunosato, Tsukuba 305-8687, Japan; E-Mails: otatsu@ffpri.affrc.go.jp (T.O.); kagawa@ffpri.affrc.go.jp (T.K.); 4 Center for Environment, Health and Field Sciences, Chiba University, 6-2-1 Kashiwanoha, Kashiwa, Chiba 277-0882, Japan

**Keywords:** terraced paddy field, physiological and psychological response, stress reduction, health benefit of rural environment

## Abstract

Despite an increasing attention and public preference for rural amenities, little evidence is available on the health benefits of a rural environment. In this study, we identified physiological and psychological benefits of exposure to a rural environment using multiparametric methods. Twelve young male adults participated in a 3-day field experiment (mean ± standard deviation age, 22.3 ± 1.3 years). Sleeping environment, diet program, physical activities, and other factors possibly affecting physiological responses were controlled during experiment period. For all participants, salivary cortisol concentration, heart rate variability, and blood pressure were measured at rural and urban field sites. Self-evaluation questionnaires were administered to analyze the psychological states in two different environments. Volatile compounds in the air were also analyzed to investigate air quality. The data were compared between rural and urban environments. The data showed that exposure to a rural environment reduced stress hormone secretion and sympathetic nervous activity and increased parasympathetic nervous activity. Short-term exposure to a rural environment also improved mood states. Our findings indicate that exposure to a rural environment effectively reduced physiological stress and enhanced psychological well-being.

## 1. Introduction

More than 50% of the world’s population currently lives in cities [1]. Urbanization is one of the most fundamental characteristics in environmental changes, involving a broad range of environmental issues such as landscape change [2], air pollution [3], and climate warming [4]. Urbanization has often been regarded as a potential health risk factor in the field of environmental health [5]. To date, an increasing number of studies have shown negative health effects of exposure to urban stimulations in urban areas [6,7,8]. WHO (2010) [9] points out that urban environments tend to discourage physical activity because of a variety of factors, including high-volume traffic, heavy use of motorized transportation, and poor air quality. Recent studies have reported that urbanization is increasingly linked with chronic non communicable diseases, including mental health disorders, obesity, type II diabetes, metabolic syndrome, and cardiovascular disease [10,11,12,13,14,15,16], which is partly associated with nutritional transition in modern society [17].

On the other hand, increasing attention has been given to the health benefits of exposure to natural environments [18]. Since the late 20th century, a substantial body of research has illustrated the positive effects of exposure to natural environments on varied psychological parameters, including stress reduction, mood state promotion, recovery from fatigue, improved attention, and enhanced job satisfaction [19,20,21,22,23,24,25,26,27,28]. Compared with physical activity in an urban setting, physical activity in a rural setting is known to be more advantageous from the aspect of restoration [29,30]. Epidemiological investigations have shown that contact with natural environments is positively associated with health parameters, such as mental health [31], reduced health inequality [32], and longevity in urban seniors [33]. In addition, recent physiological studies have provided strong evidence supporting direct health benefits of exposure to forest environments by investigating the central nervous activity [34], autonomic nervous activity [35,36,37,38], endocrine activity [34,36,37,38], and immune function [39,40].

Social needs for rural amenities are rapidly growing with rising living standards, added leisure, and recreational activities, and there is an increasing interest in health promotion [41,42]. Health concerns regarding city living [8,43] stress the importance of rural amenities from the perspective of health promotion of urban dwellers. Rural amenities have become one of the most critical factors in the recent trend of rural migration in US [44]. Recent studies have provided evidence supporting viewing rural landscapes may provide positive health benefits [45]. One study reported that walking in a rural setting was more advantageous to mood and mindset than walking in an urban setting [46].

Despite increasing attention and public preference for rural amenities [47,48], there is still insufficient scientific evidence supporting the direct health benefits of rural environments. To address this issue, measuring human physiological responses of subjects exposed to real environmental stimuli would be the most valid method. This field approach has been applied in research on the benefits of forests and has provided important evidence that could not be verified in indoor experiments [34,39,49]. In addition, compared with an indoor approach, the field approach increases the ability to generalize study effects [50]. Therefore, the aim of this study was to measure physiological responses associated with exposure to a rural environment to investigate the potential acute health benefits in urban dwellers.

## 2. Experimental Section

### 2.1. Subjects and Study Sites

The subjects were 12 young adult male students recruited from a local university. The mean age of the participants was 22.3 ± 1.3 years (mean ± standard deviation). In the recruiting process, the following exclusion criteria were used: past and current mental disorders, cardiovascular or allergic diseases, and smoking or drinking habits. Before the study, the aims and protocol of the study were concretely explained, and written informed consent was obtained from every participant. The names of the participants were randomly coded. This study was conducted after obtaining approval from the Ethics Committee of the Center for Environment, Health and Field Sciences, Chiba University.

**Figure 1:**
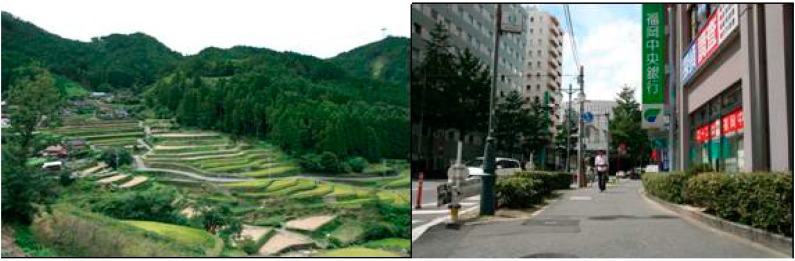
Rural landscape with terraced paddy field in Ukiha City (**Left**) and urban landscape with traffic and buildings in Fukuoka City (**Right**) in southwestern Japan.

To examine the physiological and psychological effects when exposed to real rural environments, a traditional paddy field landscape in Ukiha City in southern Japan was selected as the study site (Figure 1). The terraced paddy field is one of the typical rural landscapes in many Asian countries and has high scenic value. As a control, an urban site around the Hakata station, which is one of the largest railway terminals in southern Japan, was selected because the railway terminal is the most frequently used facilities in Japan. The field study was conducted in autumn, and the weather was generally pleasant throughout the study.

### 2.2. Experimental Design

To examine the acute effects of contact with traditional rural landscapes on urban dwellers, human physiological and psychological responses at field sites were measured. Throughout the experiment, the time schedule, meal and water intake, sleeping environment, and physical activity of the participants were controlled to exclude variables, except for environmental stimuli, that may have affected the subjects’ physiological conditions. The field experiment was conducted for 3 days, and all participants stayed in the same type of single room in a hotel during the experimental period. They were switched to a controlled schedule to control their physical activities. The participants’ meals were provided according to a scheduled menu throughout the experiment. Breakfast, lunch, and dinner were prepared to provide the equal nutrition and calories to each participant. Intake of caffeine, including coffee, tea, and soft drinks, as well as smoking and drinking were prohibited. During the daytime, all participants participated in the field experiment or remained in a waiting and read books. After completing the field experiment, all participants stayed at a hotel and spent time watching television or reading books. They were prohibited from going out at night, and the sleeping time was between 10 PM and 6 AM. Other factors that could possibly influence the physiological or psychological responses, such as hot spa bathing and use of cell phones and music players, were also controlled.

On the first day of the experiment, all participants were gathered in a prepared room, and a general explanation of this study was provided. Then, the participants previsited the field sites in the rural and urban landscapes where the physiological and psychological measurements would be made so that the participants could easily understand the experimental process. A previsit is important for reducing data errors and clearly capturing the effects of environmental stimuli because it eliminates the psychological tension caused by the first experience. On the second day, all participants were randomly divided into two groups and allocated to rural or urban sites. The first set of physiological and psychological data was obtained immediately after waking up at the hotel as a baseline (Table 1). After breakfast, all participants traveled to each designated field site by car. Variations in the travel time were minimized by adjusting the moving routes, irrespective of the study site. At each site, measurements were made on one person at a time. Each participant rested in a seated position to exclude the effects of physical activity and stabilize the physiological condition before measurements. Then, the second set of data was measured during the pre-exposure period. According to the protocol, each participant viewed the rural or urban landscape for 15 min, during which a participant was fully exposed to real environmental stimuli, such as scene, sound, smell, and air quality. After exposure, the third set of data was collected during the post-exposure period. For heart rate variability (HRV), the data were recorded continuously throughout the exposure. On the third day, each participant was assigned to another field site, and the data were obtained by following the same protocol as used on the second day.

**Table 1:** Baseline values of the subjects in rural and urban environments.

	Rural		Urban		Differences
	Mean	SE	Mean	SE	
**Physiological parameters**
Pulse rate(bpm)	59.1	3.0	61.5	3.6	ns
SBP(mmHg) ^a^	116.0	2.1	122.2	3.5	ns
DBP(mmHg) ^a^	61.7	1.9	64.1	2.0	ns
ln(HF)	6.5	0.2	6.1	0.4	ns
ln(LF/HF)	−2.3	0.7	−3.1	0.8	ns
**Psychological parameters**
SD					
Comfortable feeling	2.5	0.5	1.5	0.5	ns
Soothed feeling	1.9	0.7	2.3	0.7	ns
Natural feeling	−1.2	1.0	−1.0	0.8	ns
Refreshed feeling	47.5	5.0	52.7	4.1	ns
POMS					
Tension-anxiety	47.7	3.5	44.6	3.8	ns
Depression	48.7	3.3	49.1	3.9	ns
Anger-hostility	44.3	3.0	43.9	2.7	ns
Fatigue	48.1	4.0	47.1	3.7	ns
Confusion	45.4	2.7	47.3	3.8	ns
Vigor	45.4	2.7	47.3	3.8	ns
Total mood disturbance	191.6	17.0	189.5	18.5	ns

Notes: ^**a**^ SBP, systolic blood pressure; DBP, diastolic blood pressure.

### 2.3. Measurement

#### 2.3.1. Physiological Parameters

As indices of autonomic nervous activity, systolic blood pressure, diastolic blood pressure, and pulse rate were measured using a portable blood pressure monitor (HEM-1000; Omron, Tokyo, Japan). HRV, a parameter currently used to assess sympathetic and parasympathetic activities, was measured using a portable electrocardiograph (Activtracer AC-301A; GMS, Tokyo, Japan). Autonomic functions were investigated in all measurement periods at both the rural and urban sites. As an index of endocrine activity, salivary cortisol, a reliable stress hormone that shows human stress reactions, was investigated. Saliva samples were collected using a salivette (No. 51.1534; Sarstedt, Nümbrecht, Germany), and the cortisol concentration was analyzed. The sampling method is very simple and noninvasive. Saliva samples were taken before and after exposure to the environmental stimuli and the values were compared. Saliva samples taken at the field sites were immediately placed in a freezer and sent to a laboratory (SRL Inc., Tsukuba, Japan) for analysis of cortisol levels.

#### 2.3.2. Questionnaires

Subjective evaluation methods were applied to measure the psychological responses to environmental stimuli. The semantic differential method [51] was used to explore the participants’ perceptions on the two different environments. The semantic differential scale asks the subjects to rate an impression of each environment on a 13-point scale that has two bipolar adjectives (comfortable–uncomfortable, soothed–awakened, natural–artificial) at each end. The feeling of refreshment was investigated using a questionnaire with 30 questions which had a total score range of 0–90 [52]. This questionnaire, a commonly used stress response checklist, contains multiple adjectives that are rated by subjects on a 4-point scale to ascertain the degree to which they felt refreshed. These psychological reactions were examined in all measurement periods. In addition, the shortened Japanese version of the Profile of Mood States (POMS) [53] was used to assess the following six mood dimensions on a 13-point scale: “tension–anxiety (T–A)”, “depression (D)”, “anger–hostility (A–H)”, “confusion (C)”, “vigor (V)”, and “fatigue (F)”. The POMS tests, a widely used psychological rating scale applied to assess transient mood states, were administered during the pre- and post-exposure periods.

#### 2.3.3. Air Quality Analysis

While considering the health effects of natural environments, less attention has been given to volatile organic compounds (VOCs), which may have affected the health outcomes of urban dwellers. The air samples were taken to analyze VOCs in the atmosphere of the two study sites. Rural samples were taken near the terraced paddy field at an elevation of 400–450 m located in Ukiha City, and the urban samples were taken in the Fukuoka City area in southern Japan. The organic constituents in the air were trapped in glass cartridges (PEJ-02; Supelco, Bellefonte, PA, USA), which were filled with an adsorbent (140 mg of Carboxen 1000 and 100 mg of Carbopac B, 60–80 mesh). The adsorbent tubes were conditioned three times for 30 min at 295 °C in a helium gas flow of approximately 10 mL/min. A total amount of 147 L of rural air was sampled for 24.5 h, and a total of 39 L of city air was sampled for 6.5 h (sampling pump: MP-Σ30; Shibata, Tokyo, Japan) 1.2-m above the ground.

An ATD 400 automatic thermodosorption (PerkinElmer, Waltham, MA, USA) device coupled with gas chromatography–mass spectrometry (GC–MS) was used for analysis. The trapped volatiles in the adsorbent tube (PEJ-02) were preheated at 240 °C for desorption of the volatiles from the adsorbent in a heater block with a heat controller for 15 min and collected into a cold trapping tube (Air-monitoring tube; PerkinElmer) at −30 °C. Then, the volatiles were flushed into the gas chromatograph from a cold trapping tube in a heater block with a heat controller at 300 °C for 15 min.

The components were identified by GC–MS analysis. Analytical runs were performed on a Hewlett-Packard 5973/6890 GC–MS (Hewlett Packard, Wilmington, DE, USA) equipped with selected ion monitoring (SIM) functions. The chromatographic conditions were: GC analytical column, HP-5MS (30-m length, 0.25-mm i.d.); temperature program, 40 °C (15 min), 40 °C (at 4 °C/min), 180 °C (15 min), 180 °C (at 5 °C/min), 280 °C (15 min), 280 °C; carrier gas, helium at 1.2 mL/min. Mass spectra were obtained at 70 eV, and peak identity was confirmed by comparison with standards. Because the monoterpene concentrations in the atmosphere were too low to record mass spectra, SIM was applied. The ions used by SIM were m/z 68, 93, and 136 because these are typical of monoterpene mass spectra. The concentrations of monoterpenes in the samples were usually determined from the peak heights of the SIM chromatogram at m/z 93 using a calibration curve prepared from standard solutions.

### 2.4. Data Analysis

HRV data were assessed at various frequency bands using an HRV software tool (MemCalc/win; GMS, Tokyo, Japan). In a continuously recorded data, interbeat (R–R) intervals were obtained for a 1-min segment using the maximum entropy method. In this study, the two major spectral components of HRV, the variances of the low-frequency (LF; 0.04–0.15 Hz) band and high-frequency (HF; 0.15–0.4 Hz) band, were calculated [54]. The HF data can be used as an index of parasympathetic nervous activity, and the LF/HF ratio can be used as an index of sympathetic nervous activity. HRV values were expressed as the natural logarithm (ln). In the POMS test, the T-score was used for analysis. Total mood disturbance (TMD) was calculated by summing the five negative mood dimensions and subtracting the vigor score. One of the 12 participants retired in the middle of the experiment, and a total of 11 samples were used for data analysis.

Comparisons between rural and urban data were performed for all parameters. For comparisons of the physiological data, a paired t-test was applied for each data set. The Wilcoxon signed-rank test was used to compare psychological data. Statistical analysis was performed by using Microsoft Excel (Microsoft Inc. Redmond, WA, USA), and subjective data were processed using SPSS 21.0 (IBM-SPSS Inc, Chicago, IL, USA). The statistical differences were considered significant at *p* < 0.05. All values were expressed as the mean ± standard error (SE).

## 3. Results

### 3.1. Physiological Parameters

**Figure 2:**
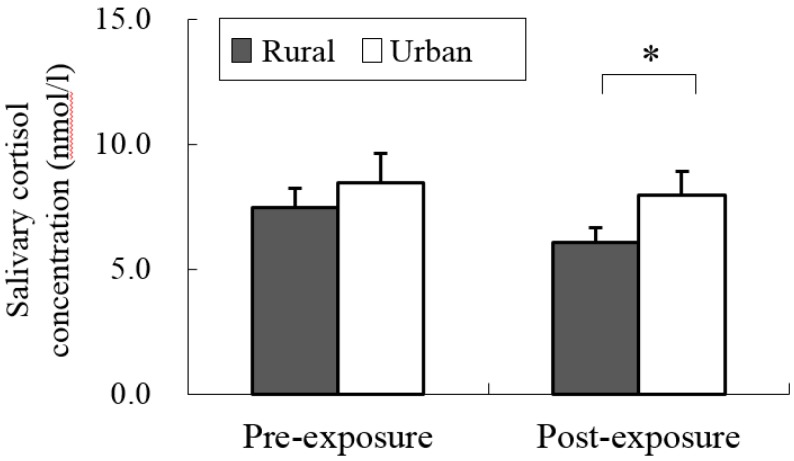
Comparison of salivary cortisol concentrations in participants at pre- and post- exposure sessions between rural and urban environments. Mean ± SE; N = 11; * *p* < 0.05; paired *t*-test.

**Figure 3:**
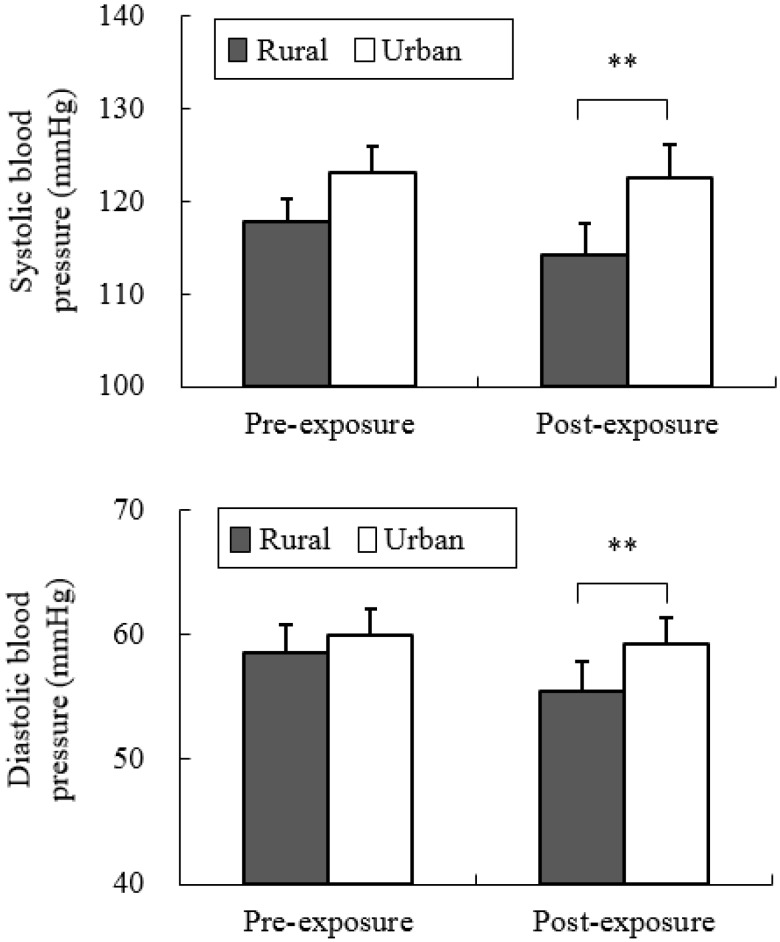

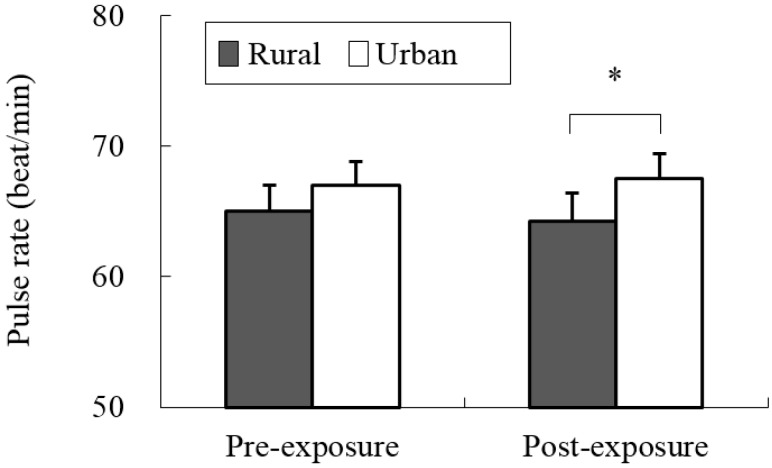
Comparison of systolic (top) and diastolic blood pressures (middle) and pulse rate (bottom) between the rural and urban at pre- and post- exposure sessions. Mean ± SE; N = 11; * *p* < 0.05; ** *p* < 0.01; paired *t*-test.

**Figure 4:**
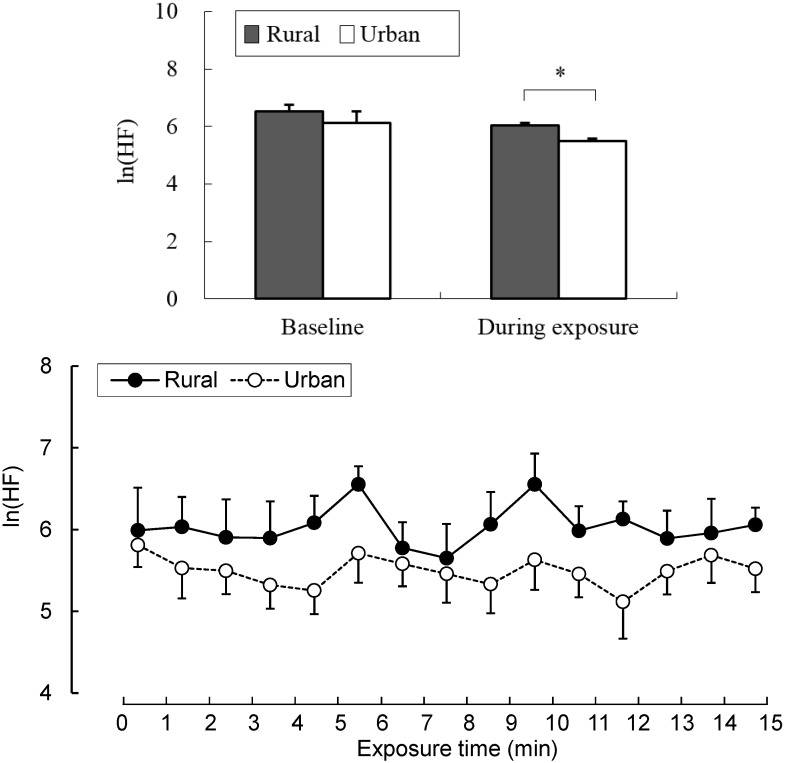
Comparison of the natural logarithm of the high frequency value in heart rate variability between rural and urban exposures (top) and of the 1-min fluctuations of the values (bottom) during exposure. Mean ± SE; N = 11; * *p* < 0.05; paired *t*-test.

**Figure 5:**
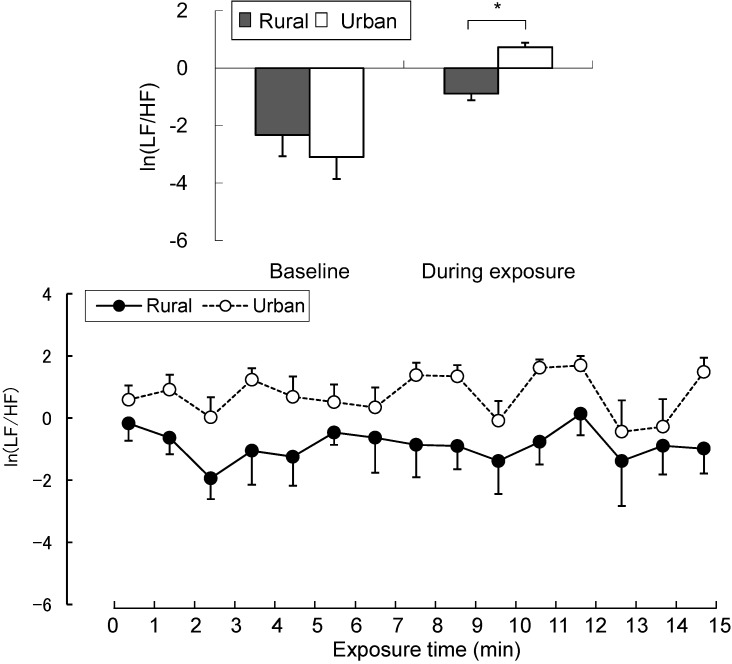
Comparison of the natural logarithm of high frequency/low frequency ratio in heart rate variability between rural and urban exposures (top) and of 1-min fluctuations of the value (bottom) during exposure. Mean ± SE; N = 11; * *p* < 0.05; paired *t*-test.

Our data revealed different physiological effects of exposure to the rural and urban environments. In the analysis of salivary cortisol concentration, a significant difference was found between the two environments. When exposed to the rural environment, the level of salivary cortisol (6.07 ± 0.57 nmol/L) was significantly decreased relative to that from urban exposure (7.95 ± 0.96 nmol/L; *p* < 0.05; Figure 2), although no significant differences were observed in the pre-exposure period (rural, 7.47 ± 0.77 nmol/L; urban, 8.45 ± 1.17 nmol/L). Significant differences were identified for the parameters of autonomic nervous activity. Systolic blood pressure after short-term exposure to real environments was significantly decreased in the rural environment (114.1 ± 3.4 mmHg) relative to that in the urban environment (122.6 ± 3.4 mmHg; *p* < 0.01; Figure 3 top), although no significant differences were observed in the baseline (rural, 116.0 ± 2.1 mmHg; urban, 122.2 ± 3.5 mmHg) and pre-exposure periods (rural, 117.8 ± 2.4 mmHg; urban, 123.0 ± 2.9 mmHg). Diastolic blood pressure in the post-exposure period was significantly lower in the rural environment (55.4 ± 2.4 mmHg) than in the urban environment (59.3 ± 2.1 mmHg; *p* < 0.01; Figure 3 middle), with no significant differences in the baseline (rural, 61.7 ± 1.9 mmHg; urban, 64.1 ± 2.0 mmHg) and pre-exposure periods (rural, 58.5 ± 2.2 mmHg; urban, 60.0 ± 2.1 mmHg). Pulse rate was significantly lower after exposure to the rural (64.3 ± 2.2 beats/min), compared to the urban environments (67.5 ± 1.9 beats/min; *p* < 0.05; Figure 3 bottom), although no significant differences were observed in the baseline (rural, 59.1 ± 3.0 beats/min; urban, 61.5 ± 3.6 beats/min) and pre-exposure periods (rural, 65.0 ± 2.0 beats/min; urban, 67.0 ± 1.9 beats/min) between the two environments. In the analysis of HRV data, the mean 15-min ln(HF) values that reflected parasympathetic nervous activity were significantly higher in the rural environment (6.03 ± 0.09) than in the urban environment (5.49 ± 0.08; *p* < 0.01; Figure 4 top). The 1-min analysis of ln(HF) showed that the values were persistently higher in the rural environment than in the urban environment (Figure 4 bottom). However, there were no significant differences in the baseline values between the rural and urban environments. On the other hand, the mean 15-min ln(LF/HF) values, which reflected sympathetic nervous activity, were significantly lower in the rural environment (−0.89 ± 0.23) than in the urban environment (0.73 ± 0.15; *p* < 0.01; Figure 5 top), although no significant differences were observed in the baseline values. The 1-min analysis of ln(LF/HF) showed that the values were persistently lower in the rural environment than in the urban environment during 15 min of exposure (Figure 5 bottom).

### 3.2. Psychological Parameters

**Figure 6:**
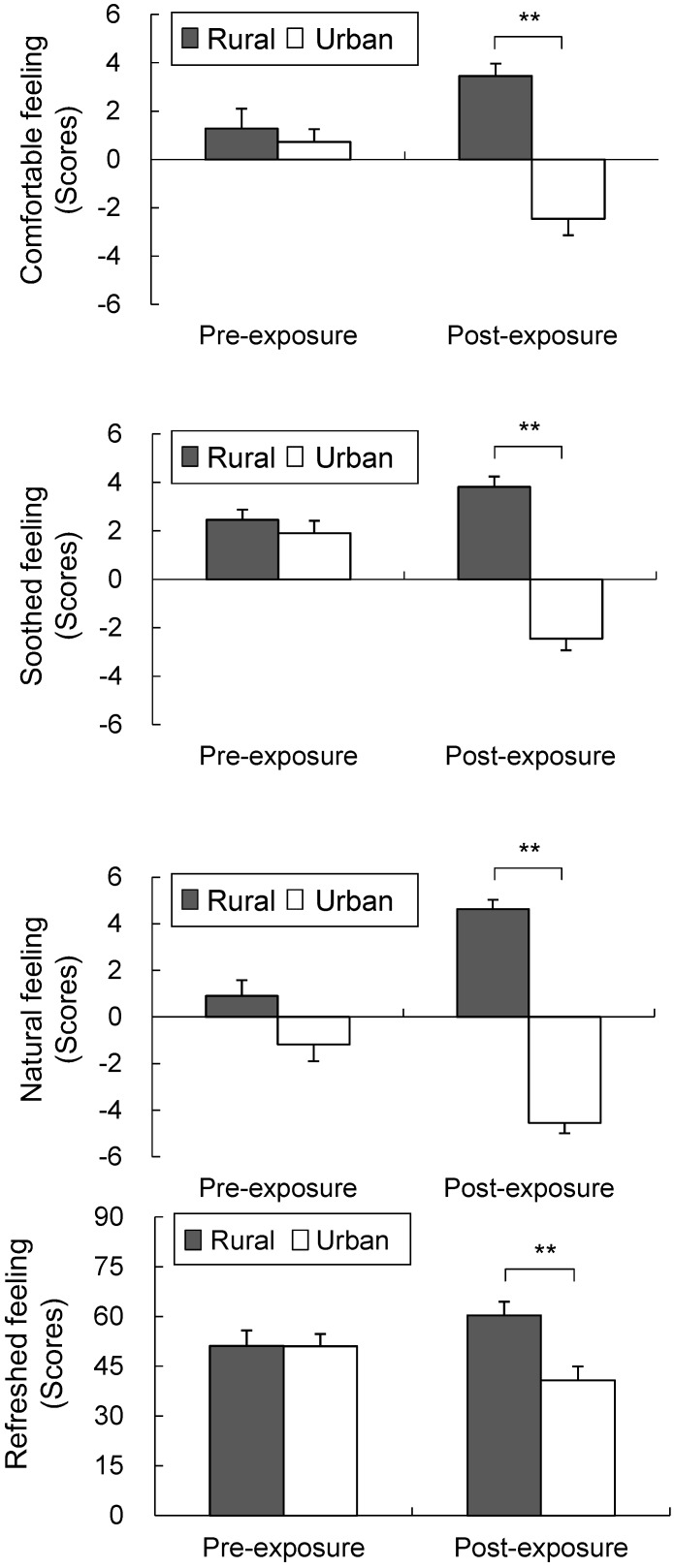
Comparison of the perceived comfortable (top), soothed (upper middle), natural (lower middle), and refreshed feelings (bottom) between the rural and urban environments at pre- and post- exposure sessions. Mean ± SE; N = 11; ** *p* < 0.01; Wilcoxon signed-rank test.

**Figure 7:**
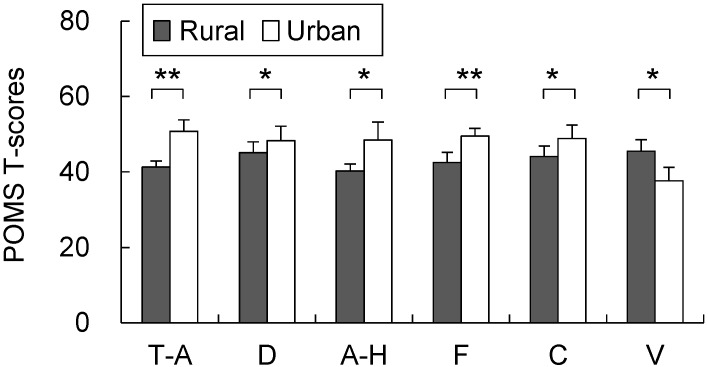
Comparison of the Profile of Mood States (POMS) scores after exposure to the rural and urban landscapes. Mean ± SE; N = 11; * *p* < 0.05; ** *p* < 0.01; Wilcoxon signed-rank test. T–A, tension–anxiety; D, depression; A–H, anger–hostility; F, fatigue; C, confusion; V, vigor.

Compared with the urban environment, the rural environment had significantly positive effects on the participants’ feeling and mood states. The participants responded that they felt significantly more comfortable (rural, 3.5 ± 0.5; urban, −2.5 ± 0.7; *p* < 0.01; Figure 6 top), more soothed (rural, 3.8 ± 0.4; urban, −2.5 ± 0.5; *p* < 0.01; Figure 6 middle top), more natural (rural, 4.6 ± 0.4; urban, −4.5 ± 0.5; *p* < 0.01; Figure 6 middle bottom), and more refreshed (rural, 60.4 ± 4.1; urban, 40.7 ± 4.2; *p* < 0.01; Figure 6 bottom) in the rural environment than in the urban environment, although no significant differences were observed between the two in the baseline and pre-exposure periods. In the POMS analysis (Figure 7), significant differences were observed during the post-exposure period between the rural and urban environments, respectively, for all of the subscale scores including those for T–A (41.3 ± 1.6; 50.7 ± 3.1; *p* < 0.01), D (45.1 ± 2.9; 48.3 ± 3.8; *p* < 0.05), A–H (40.3 ± 1.8; 48.5 ± 4.8; *p* < 0.05), V (45.5 ± 2.7; 37.6 ± 2.1; *p* < 0.05), F (42.5 ± 2.8; 49.5 ± 3.6; *p* < 0.05), C (44.1 ± 3.0; 48.8 ± 3.6; *p* < 0.05), and TMD (167.6 ± 12.7; 208.1 ± 16.1; *p* < 0.01). However, no significant differences were observed in the baseline period values between the rural and urban environments, respectively: T–A (47.7 ± 3.5; 44.6 ± 3.8), D (48.7 ± 3.3; 49.1 ± 3.9), A–H (44.3 ± 3.0; 43.9 ± 2.7), V (42.5 ± 2.7; 42.5 ± 2.6), F (48.1 ± 4.0; 47.1 ± 3.7), C (45.4 ± 2.7; 47.3 ± 3.8), and TMD scores (191.6 ± 17.0; 189.5 ± 18.5).

### 3.3. Air Quality Analysis

There were considerable differences in the composition of volatile organic compounds between the rural and urban air samples. In the rural samples, ambient VOCs of biogenic origins were abundant, whereas in the urban samples, VOCs were mainly of anthropogenic origins with several contributions from motor vehicles. Twelve terpenoids were identified in the rural air samples (Table 2A). The main monoterpenes present in measurable amounts were α-pinene, camphene, D-limonene, and isoprene. On the other hand, toluene and other aromatic compounds were dominant in the urban air samples, with contributions from vehicle exhaust and industry processes (Table 2B). Urban air was also characterized by various solvents including ethyl acetate, chloroform, dichloromethane, xylene, 1,2,4-trimethylbenzene, and *n*-hexane.

**Table 2:** (**A**) Terpenoids identified in rural air (Ukiha City, Japan) and (**B**) pollution products and terpenoids identified in urban air (Fukuoka City, Japan). (**A**) Rural air analysis; (**B**) Urban air analysis.

**A**		
	**Compounds**	**Concentrations (ng/m^3^)**
	Isoprene	28.0
	Tricyclene	13.1
	α-Pinene	677.3
	Camphene	107.7
	β-Pinene	15.5
	Myrcene	22.1
	δ-3-Carene	16.7
	ρ-Cymene	16.8
	D-Limonene	53.4
	γ-Terpinene	10.1
	α-Terpinolene	3.7
	Bornyl acetate	2.6
These values are determined by absolute calibration method using toluene.		
**B**		
**Groups**	**Compounds**	**Concentrations (ng/m^3^)**	
Alkanes	n-Hexane	3414
	2,4-Dimethylpentane	421
	iso-Octane	450
	Heptane	678
	Octane	188
	Nonane	459
	Decane	622
	Undecane	344
	Dodecane	42
	Tridecane	nd
Aromatic compounds	Benzene	1481
	Toluene	14,104
	Ethylbenzene	1771
	o, m, p-Xylene	2873
	Styrene	139
	m-Ethyltoluene	1024
	p-Ethyltoluene	424
	1, 3, 5-Trimethylbenzene	412
	o-Ethyltoluene	374
	1, 2, 4-Trimethylbenzene	1870
	1, 2, 3-Trimethylbenzene	337
	1, 2, 4, 5-Tetramethylbenzene	35
Terpenes	α-Pinene	80
	β-Pinene	5
	D-Limonene	nd
Halogenated compounds	Dichloromethane	5345
	Chloroform	1686
	1, 2-Dichloroethane	7
	Trichloroethylene	16
	1, 2-Dichloropropane	nd
	Tetrachloroethylene	165
	p-Dichlorobenzene	294
Esters	Ethyl acetate	5722
	Butyl acetate	930
Alcohols	Nonanol	520
	Decanol	125
	Ethanol	nd
Aldehyde-ketone	Acetone	nd
	Methylethylketone	nd
	Methylisobutylketone	111
These values are determined by absolute calibration method using toluene.		

nd: not detected.

## 4. Discussion and Conclusions

The direct beneficial health effects of exposure to a rural environment relative to exposure to an urban environment were evaluated in field experiments. This may be the pioneer study to show that terraced paddy fields, a traditional agricultural landscape in many Asian countries, can be a health promoter for modern urban dwellers.

Our data illustrates the physiological and psychological benefits of exposure to rural environments more clearly than we expected. Evidence supported that exposure to rural environments can reduce physiological stress by decreasing cortisol secretion which is associated with immune functions mediated by the natural killer cell activity. Parameters reflecting autonomic nervous function showed positive health benefits of exposure to rural environments, *i.e.*, decreased pulse rate, blood pressure, and sympathetic nervous activity (ln(LF/HF)) in addition to increased parasympathetic nervous activity (ln(HF)). Exposure to a rural environment was also found to be effective for psychological relaxation by increasing positive feelings and mood states and decreasing negative mood states.

To date, most of the studies that have explored the health-related effects of rural environments have been conducted using indoor experiments and have investigated the limited effects of isolated environmental stimulations under controlled indoor conditions [8]. Because health benefits are obtained from the combination of all environmental stimuli, including views, sounds, smells, and air quality, a field study can provide a better indication of the effects of real environments than can an indoor study [50]. Although Roe and Aspinall [46] performed field experiments to investigate the restoration effects of walking in a rural setting, their study had a limitation with respect to isolating the effects solely from the rural environments because walking activity itself can affect health parameters. In addition, the previous study did not suggest the physiological benefits of rural environments because it used psychological parameters to investigate mood and cognitive characteristics. Therefore, the present study is important because the field data clearly illustrated the health benefits of exposure to a real rural environment and potential factors that could affect the participants’ physiological outcomes, such as physical activity, diet-related conditions, and sleeping environments, were controlled.

Exposure to a real rural environment appears to be more beneficial than exposure to virtual rural environments. Stress hormone secretion investigated by measuring salivary cortisol was found to significantly decrease following rural exposure. The 1-min HRV analysis of HF and LF/HF showed persistent differences in the values throughout the 15-min exposure period between rural and urban environments. However, in a previous study conducted in a laboratory setting, the HRV effect was observed only for 5 min of rural exposure [55]. Regarding the duration of the exposure effects, this study indicated that exposure to real environmental stimuli can prolong the positive health effects relative to those for exposure to laboratory stimuli. This finding may be associated with the overall strength of the real stimuli provided by a combination of multiple environmental factors, such as views, sounds, smells, and air quality, which may induce greater health benefits than those provided by viewing isolated nature images in a laboratory. In the analysis of air quality, α-pinene was the most abundant VOC in rural area. A previous indoor study by Tsunetsugu *et al.* [56] reported that Japanese cedar scent, dominated by α-pinene compounds, can decrease systolic blood pressure and total hemoglobin concentration in the prefrontal cortex. On the basis of this previous finding, we speculated that the VOCs in the air in rural area might have affected the positive health outcomes in this study. In addition, in laboratory research, the strength of the stimuli may affect the results because human physiological and psychological responses can differ depending on how realistic the stimuli are, as observed in a recent study that used two-dimensional and three-dimensional images to investigate the prefrontal cortex and autonomic nervous activities [57].

The preference for natural environments has often been explained by the biophilia hypothesis [58], attention recovery theory [22], and psycho-evolution theory [27]. These theories mainly approach this issue from the perspective of psychology, and various psychological studies support the idea that a natural environment is positively related to stress reduction, mood state promotion, recovery from fatigue, and improved vitality [19,20,21,23,27,28,59]. However, these relationships cannot provide a sufficient explanation for the health benefit mechanism. On the basis of increasing evidence on human physiological reactions to nature in recent years, the preference for natural environments may be partly explained by biological reactions to maintain homeostatic equilibrium [49]. Growing evidence from experimental studies supports the idea that exposure to natural environments positively affects the central nervous system [8,34,55], sympathetic and parasympathetic nervous systems [35,36,38,55], endocrine system [34,36,37,38,39], and immune systems [39,40]. For example, decreased immune function associated with chronic stress and fatigue recovered to normal levels following 3 days of nature experience [39,40]. The nervous, endocrine, and immune systems are interrelated [60], which also contributes to mental health conditions, such as anxiety and depression, through neurotransmitters or hormones [61]. Therefore, this physiological evidence may help explain the fact that exposure to natural environments is associated with positive health outcomes [32,33].

Urban environments in most developed countries have been planned and managed by mainly focusing on the increase in convenience and efficiency without giving thorough consideration to the effects of urban physical environments on human health. Most of the efforts regarding urban health issues have been made to reduce the negative effects of urban pollution. In the recent years, with increasing recognition of the fact that excess artificial environmental stimulation can cause negative effects on individual and community health [7,62], more and more attention has been given to natural environments. Our data suggest that visiting a rural environment may provide an effective chance for stress reduction, particularly for urban dwellers at higher risk of stress-related health problems. However, the health benefits identified in this study are not linked to the idea that rural dwellers are healthier than urban dwellers because general health conditions are also related to many other factors, including accessibility to health care service.

Despite the insufficient population size to generalize the present findings, they were generally consistent with the findings of previous large-sample experiments performed in forest environments [49,63,64]. Furthermore, given that being raised in a rural environment lowers the prevalence of asthma and atopy among rural adolescents [65] and the risk of mental and physical health problems in adulthood [66], exposure to rural environments needs to be considered as an effective tool for management of modern health problems. A limitation of the study was that the participants knew the purpose of the study, which was a potential source of bias and may have influenced their answers to the psychological tests. Recommendations include further investigation of the evidence in a larger population size with longer exposure and the mechanism underlying the health benefits of rural environments. Close collaboration also should be undertaken among health professionals, urban and rural planners, policy makers, and other concerned interest groups to utilize exposure to rural environments as a new health promoting agent that may help reduce healthcare costs.
