# Correction: Lee, J. et al. Acute Effects of Exposure to a Traditional Rural Environment on Urban Dwellers: A Crossover Field Study in Terraced Farmland. *Int. J. Environ. Res. Public Health* 2015, *12*, 1874–1893

**DOI:** 10.3390/ijerph16245052

**Published:** 2019-12-11

**Authors:** Juyoung Lee, Bum-Jin Park, Tatsuro Ohira, Takahide Kagawa, Yoshifumi Miyazaki

**Affiliations:** 1 Korea Forest Service, Government Complex 1, 189 Cheongsa-Ro, Seo-Gu, Daejeon 302-701, Korea; lohawi@gmail.com; 2 Department of Environment and Forest Resources, Chungnam National University, 99 Daehak-ro, Yuseong-gu, Daejeon 34134, Korea; bjpark@cnu.ac.kr; 3 Forestry and Forest Products Research Institute, 1 Matsunosato, Tsukuba, Ibaraki 305-8687, Japan; otatsu@ffpri.affrc.go.jp (T.O.); kagawa@ffpri.affrc.go.jp (T.K.); 4 Center for Environment, Health and Field Sciences, Chiba University, 6-2-1 Kashiwa-no-ha, Kashiwa, Chiba 277-0882, Japan

The authors wish to add the following corrections to their paper published in the International Journal of Environmental Research and Public Health [1]. When calculating the T-score of Profile of Mood States (POMS) data, an error occurred. The following change should be made to Table 1, Figure 7 and its explanation in the published article. The change does not affect the conclusions of the article in any way. 

Table 1 should be replaced with the following:

Figure 7 should be replaced with the following figure:

Lines 10–12 on page 1880 should be replaced with the following text:

The T-score was used for the analysis of the POMS test.

The lines 24–27 in page 1883 should be changed as follows:

In the POMS analysis (Figure 7), significant differences were observed during the post-exposure period between the rural and urban environments, respectively, for all of the subscale scores including those for T–A (42.7 ± 2.3; 50.2 ± 3.8; *p* < 0.05), D (44.5 ± 2.8; 47.8 ± 4.0; *p* < 0.05), A–H (40.1 ± 1.7; 47.1 ± 4.4; *p* < 0.01), V (44.0 ± 2.3; 38.5 ± 2.4; *p* < 0.05), F (41.5 ± 2.6; 50.7 ± 4.0; *p* < 0.01), and C (45.6 ± 2.2; 50.9 ± 3.9; *p* < 0.05). However, no significant differences were observed in the baseline period values between the rural and urban environments, respectively: T–A (46.7 ± 3.2; 44.7 ± 4.4), D (46.8 ± 3.3; 47.2 ± 3.0), A–H (43.8 ± 2.8; 41.5 ± 1.9), V (43.1 ± 2.6; 40.9 ± 2.6), F (47.4 ± 3.9; 46.4 ± 4.2), and C (47.5 ± 2.7; 49.4 ± 3.8).

We apologize for any inconvenience caused to the readers by this error.

## Figures and Tables

**Figure 7:**
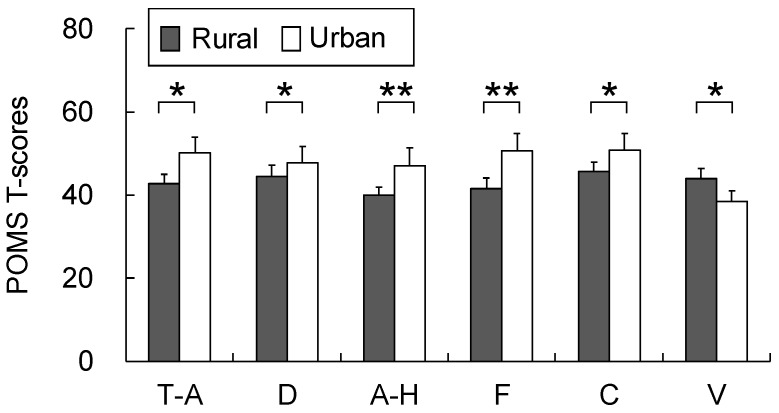
Comparison of the Profile of Mood States (POMS) scores after exposure to the rural and urban landscapes. Mean ± SE; N = 11; * *p* < 0.05; ** *p* < 0.01; Wilcoxon signed-rank test. T–A, tension–anxiety; D, depression; A–H, anger–hostility; F, fatigue; C, confusion; V, vigor.

**Table 1:** Baseline values of the subjects in rural and urban environments.

	Rural		Urban		Differences
	Mean	SE	Mean	SE	
**Physiological parameters**					
Pulse rate(bpm)	59.1	3.0	61.5	3.6	ns
SBP(mmHg) ^a^	116.0	2.1	122.2	3.5	ns
DBP(mmHg) ^b^	61.7	1.9	64.1	2.0	ns
ln(HF)	6.5	0.2	6.1	0.4	ns
ln(LF/HF)	−2.3	0.7	−3.1	0.8	ns
**Psychological parameters**					
SD					
Comfortable feeling	2.5	0.5	1.5	0.5	ns
Soothed feeling	1.9	0.7	2.3	0.7	ns
Natural feeling	−1.2	1.0	−1.0	0.8	ns
Refreshed feeling	47.5	5.0	52.7	4.1	ns
POMS					
Tension-anxiety	46.7	3.2	44.7	4.4	ns
Depression	46.8	3.3	47.2	3.0	ns
Anger-hostility	43.8	2.8	41.5	1.9	ns
Fatigue	47.4	3.9	46.4	4.2	ns
Confusion	47.5	2.7	49.4	3.8	ns
Vigor	43.1	2.6	40.9	2.6	ns

Notes: ^a^ SBP, systolic blood pressure; ^b^ DBP, diastolic blood pressure.
